# Spontaneous uterine rupture after myomectomy in patients during pregnancy: Clinical cases in a single university center

**DOI:** 10.1002/ijgo.70592

**Published:** 2025-10-16

**Authors:** Alessandra Brenta, Elena Cesari, Susanna Bonato, Valeria Maria Savasi

**Affiliations:** ^1^ Obstetrics and Gynecology Unit, Buzzi Children's Hospital University of Milan Milan Italy; ^2^ Department of Biomedical and Clinical Sciences, Obstetrics and Gynecology Unit, Buzzi Children's Hospital University of Milan Milan Italy

**Keywords:** labor, myomectomy, uterine rupture

## Abstract

Uterine rupture is an infrequent yet sometimes fatal complication of a subsequent vaginal birth attempt following a previous uterine surgery. We have chosen to write about spontaneous uterine ruptures following myomectomy due to the scarcity of data on this subject, stemming from the limited number of reported cases. Furthermore, with the increasing trend of advanced maternal age, there is a growing cohort of pregnant women with a history of myomectomy, thereby presenting a timely opportunity to examine this phenomenon in greater depth. A total of 28 studies reporting pregnancies after prior myomectomy, resulting in 3.502 viable (≥24 weeks) deliveries, were reviewed. The overall incidence of uterine rupture after myomectomy of 0.6%, comparable with those reported in other reviews. Our review confirmed that the incidence of uterine rupture is very low, 0.4%, in the group of women who experienced a trial of labor after myomectomy (TOLAM). In particular, the difference of incidences of uterine rupture before or during labor was not statistically significant. Therefore, uterine rupture may not be significantly influenced by a TOLAM and so this option could be considered in pregnant women as feasible and relatively safe. This study presents three medical cases that occurred at our institution in 2023 of pregnant patients who had undergone surgery for myomectomy and experienced uterine rupture out of labor. The first is a clinical case of a 42‐year‐old woman affected by endometriosis who had undergone laparoscopic myomectomy 1 year before conception. The actual pregnancy was conceived by intracytoplasmatic sperm injection (ICSI). The patient presented at 22^+4^ weeks' gestation to the emergency department (ED) for abdominal pain. On hospital presentation, transabdominal ultrasound evidenced a single fetus, with fetal heart rate 163 beats/min and free fluid in the Morrison's pouch with a blood clot at the uterine fundus. Abdominal computed tomography (CT) scan with and without contrast was performed due to the unclear origin of the hemoperitoneum. CT scan revealed abundant abdominal free fluid, especially perihepatic (3 cm), in the left hypochondrium (2 cm), parieto‐colic gutter and anterior the uterus, without contrast spreading; the uterus had inhomogeneous density and profiles. One hour after hospitalization, the patient was admitted to the operating room: a 10 cm fundal uterine rupture with protruding amniotic sac was present. The second is a clinical case of a 32‐year‐old woman who had undergone laparoscopic myomectomy 23 months before conception. An intramural myoma 6 cm in diameter was located on the posterior wall of the uterus. The patient conceived spontaneously 23 months later. The woman presented at 36^+3^ weeks' gestation to the ED for irregular uterine contractions (1 uterine contraction every 10–15 min). Three hours after admission, irregular uterine contractions were still present (1 uterine contraction every 10–15 min): the patient was thereby hospitalized. One hour after hospitalization, the patient reported a prolonged contraction and the transabdominal ultrasound check evidenced fetal bradycardia. An immediate cesarean section was performed, showing a massive hemoperitoneum which was promptly drained. After fetal extraction and manual removal of the placenta, a close uterine inspection was performed, showing a 15 cm uterine rupture involving the posterior wall. The third is a clinical case of a 28‐year‐old woman who had undergone laparoscopic myomectomy 2 years before conception. The patient reported that the uterine cavity was opened to remove an intramural myoma of 6 cm in diameter located on the left anterolateral wall of the uterus. She conceived spontaneously 2 years later, and the course of the pregnancy was uncomplicated. The patient presented at 31^+0^ weeks' gestation to the ED for abdominal pain. On hospital presentation, transabdominal ultrasound scan evidenced a single fetus with normal heart rate and a growing blood clot at the uterine fundus. The patient was admitted to the operating room for exploratory laparotomy, confirming a massive hemoperitoneum. A 7 cm uterine rupture with protruding amniotic sac was present in the left posterolateral uterine wall. The surgeon hence proceeded to perform hysterotomy, amniorrhexis, fetal extraction of a fetus alive and vital, and manual removal of the placenta. The patient's uterus was surgically repaired with double layer suture. The amount of total blood loss was 1800 mL. A total of four units of packed red blood cells and two units of fresh frozen plasma were transfused. The patient recovered well and was discharged 7 days after surgery.

## 1 INTRODUCTION

Uterine rupture is an infrequent yet sometimes fatal complication of a subsequent vaginal birth attempt following a previous uterine surgery.^1^ Clinically significant uterine scar rupture is defined as a full thickness tear of the uterine wall that also includes uterine serosa (overlying peritoneum).^1^ It is associated with fetal distress, the need for an emergency cesarean section, hysterectomy or uterine repair, severe uterine bleeding, protrusion/expulsion of the placenta and/or fetus into the abdominal cavity.^1^ Rupture of an unscarred uterus is rare, usually traumatic, and its incidence decreases with improvement in obstetric practice.^2^ It may result from obstetric maneuvers such as internal version and breech extraction.^2^ Such maneuvers were practiced in the past in developing countries as obstetricians attempt to avoid cesarean section and thus minimize maternal mortality and morbidity in circumstances where the anesthetic services are suboptimal^2^ In developed countries, the improvements in medical care and, therefore, the increased use of cesarean delivery, means that traumatic rupture of an unscarred uterus during pregnancy is now more likely to be associated with external trauma such as road traffic accidents or domestic violence.^2^ Rupture of an unscarred uterus may rarely complicate, for example, an instrumental vaginal delivery, manual removal of the placenta, fetal surgery, shoulder dystocia and surgical termination of pregnancy.^3^

Rupture of the scarred uterus is more common and usually occurs after a trial of labor in a patient with a previous cesarean section.^2^ A previous uterine scar is the major risk factor for uterine rupture, and the most common scar is due to a previous cesarean section. The uterus may also be scarred as a result of a previous myomectomy, a previous perforation, or a uteroplasty.^4^

We have chosen to write about spontaneous uterine ruptures following myomectomy due to the scarcity of data on this subject, stemming from the limited number of reported cases.

Furthermore, with the increasing trend of advanced maternal age, there is a growing cohort of pregnant women with a history of myomectomy, thereby presenting a timely opportunity to examine this phenomenon in greater depth.

## 2 CLINICAL CASES

The present study describes the medical case of three pregnant patients who had undergone surgery for myomectomy and experienced uterine rupture out of labor.

We retrospectively collected the medical records of three pregnant women diagnosed with out of labor uterine rupture after myomectomy at our university center (Ospedale Vittore Buzzi—Ospedale Luigi Sacco, University of Milano). A written informed consent was obtained from the patients to use their anonymized clinical information for the purpose of this study.

The first is a clinical case of a 42‐year‐old woman affected by endometriosis who had undergone laparoscopic myomectomy 1 year before conception. Detailed documentation about the surgery was not available. Her medical history included Crohn's disease treated with Mesalazine. The patient was a gravida 5 para 0, with four spontaneous abortions (only one of them was surgically treated). The actual pregnancy was conceived by intracytoplasmatic sperm injection (ICSI). The ultrasound scans of the fetus, placenta and uterus were normal. The patient presented at 22^+4^ weeks' gestation to the emergency department (ED) for abdominal pain.

On hospital presentation, vital signs were normal (arterial blood pressure 108/65 mmHg, heart rate 95 beats/min), no abnormal uterine bleeding was identified and fetal movements were well perceived. On physical examination, the abdomen was soft but diffusely tender on deep palpation. The uterus was palpable and mildly contracted. No rebound tenderness was identified. Transvaginal ultrasound scan showed a cervix >40 mm.

Transabdominal ultrasound evidenced a single fetus, with fetal heart rate 163 beats/min and free fluid in the Morrison's pouch with a blood clot at the uterine fundus. Nasopharyngeal swab for SARS‐CoV‐2 detection performed upon admission was negative and hemoglobin was 9.8 g/dL. One hour after admission, vital signs became unstable showing hypotension (arterial blood pressure 85/50 mmHg) and the patient was hospitalized with a diagnosis of acute hemoperitoneum.

Abdominal computed tomography (CT) scan with and without contrast was performed due to the unclear origin of the hemoperitoneum.

**FIGURE 1:**
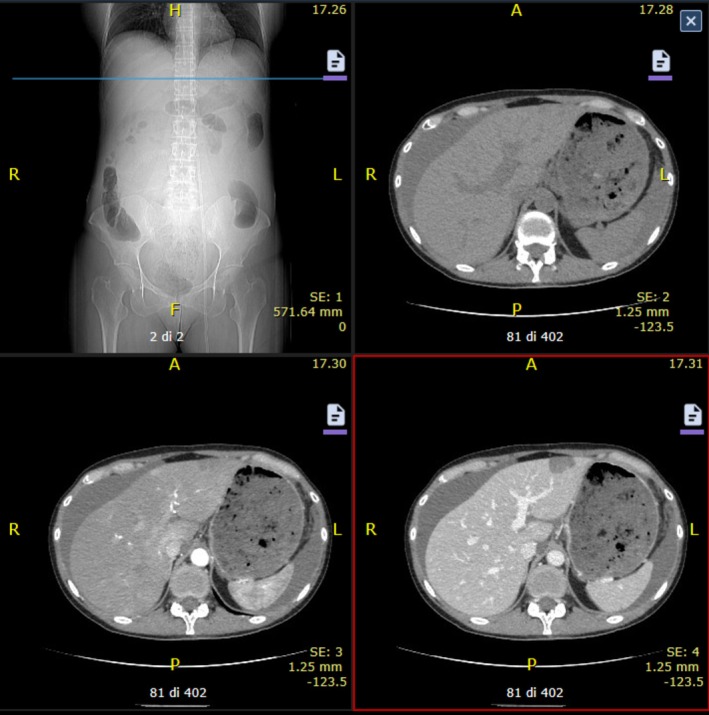
Abdominal computed tomography (CT) scan with and without contrast showing abundant abdominal free fluid, perihepatic (3 cm) and in the left hypochondrium (2 cm).

CT scan reported abundant abdominal free fluid, especially perihepatic (3 cm), in the left hypochondrium (2 cm), parieto‐colic gutter and anterior the uterus, without contrast spreading; the uterus had inhomogeneous density and profiles. The left hepatic lobe presented a focal lesion of 3 × 2 cm with a contrastographic pattern compatible with a hepatic angioma (Figure 1).

**FIGURE 2:**
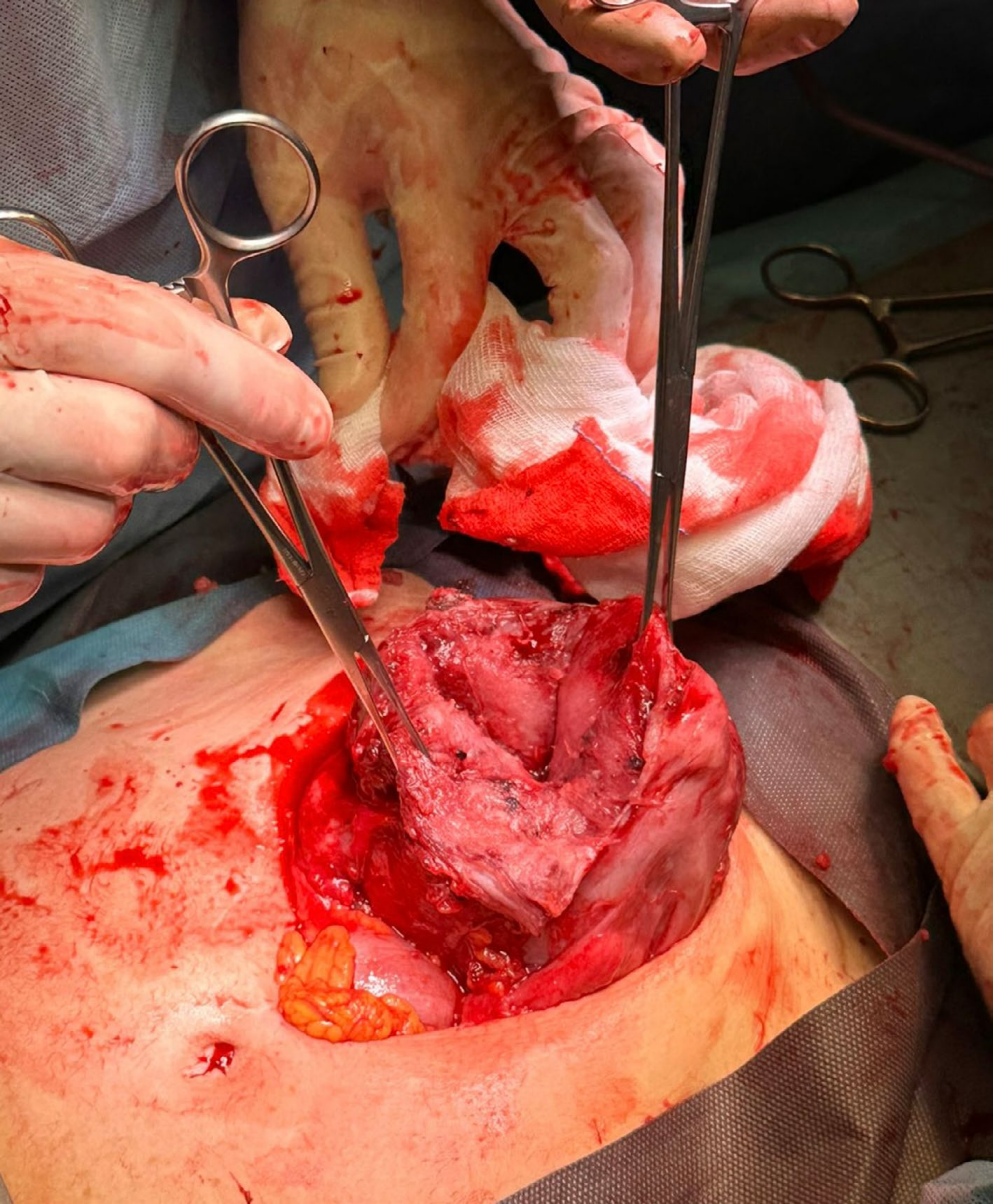
Uterine rupture identified during laparotomy.

**FIGURE 3:**
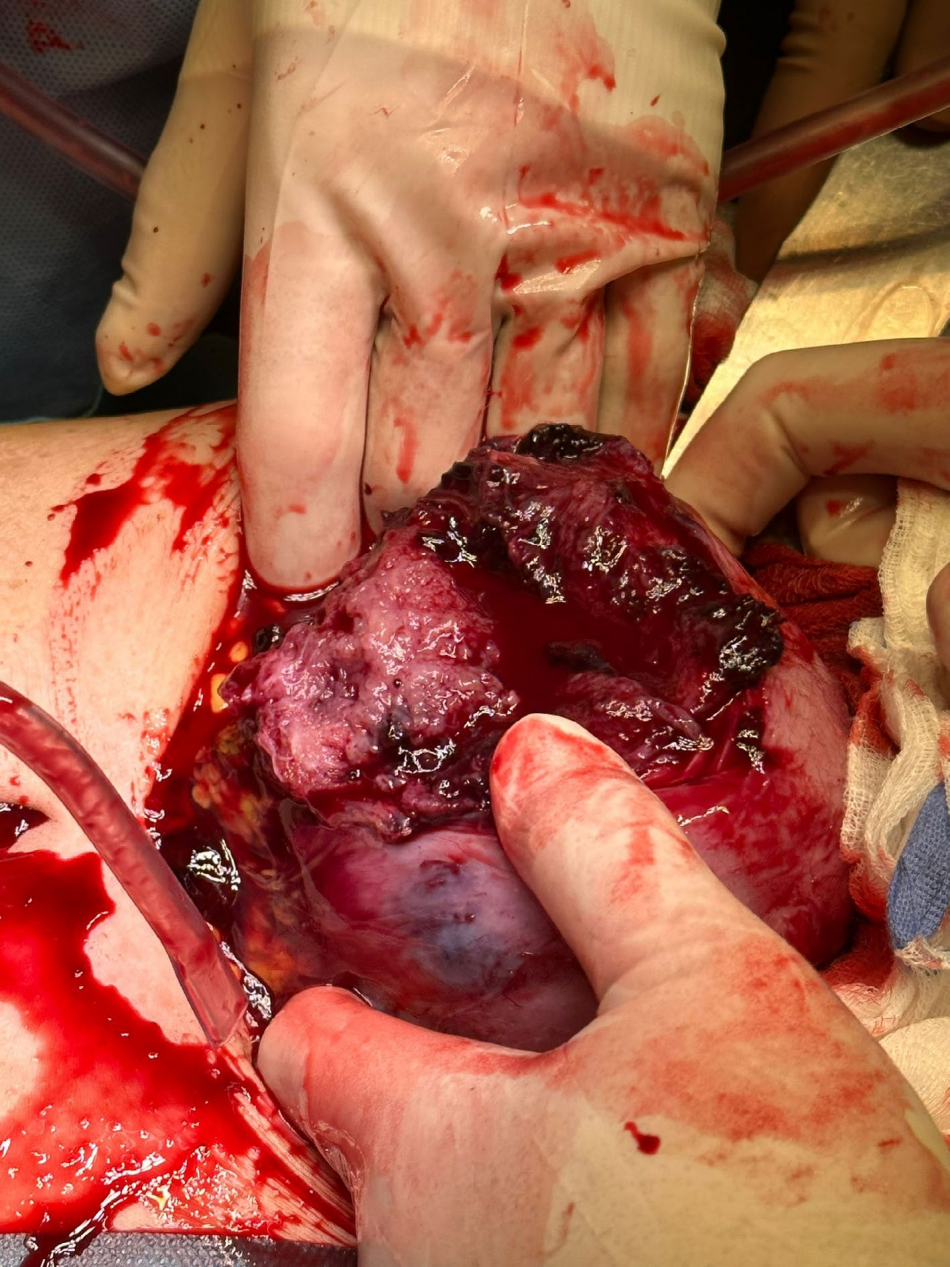
Myometrial defect reaching the endometrial cavity.

**FIGURE 4:**
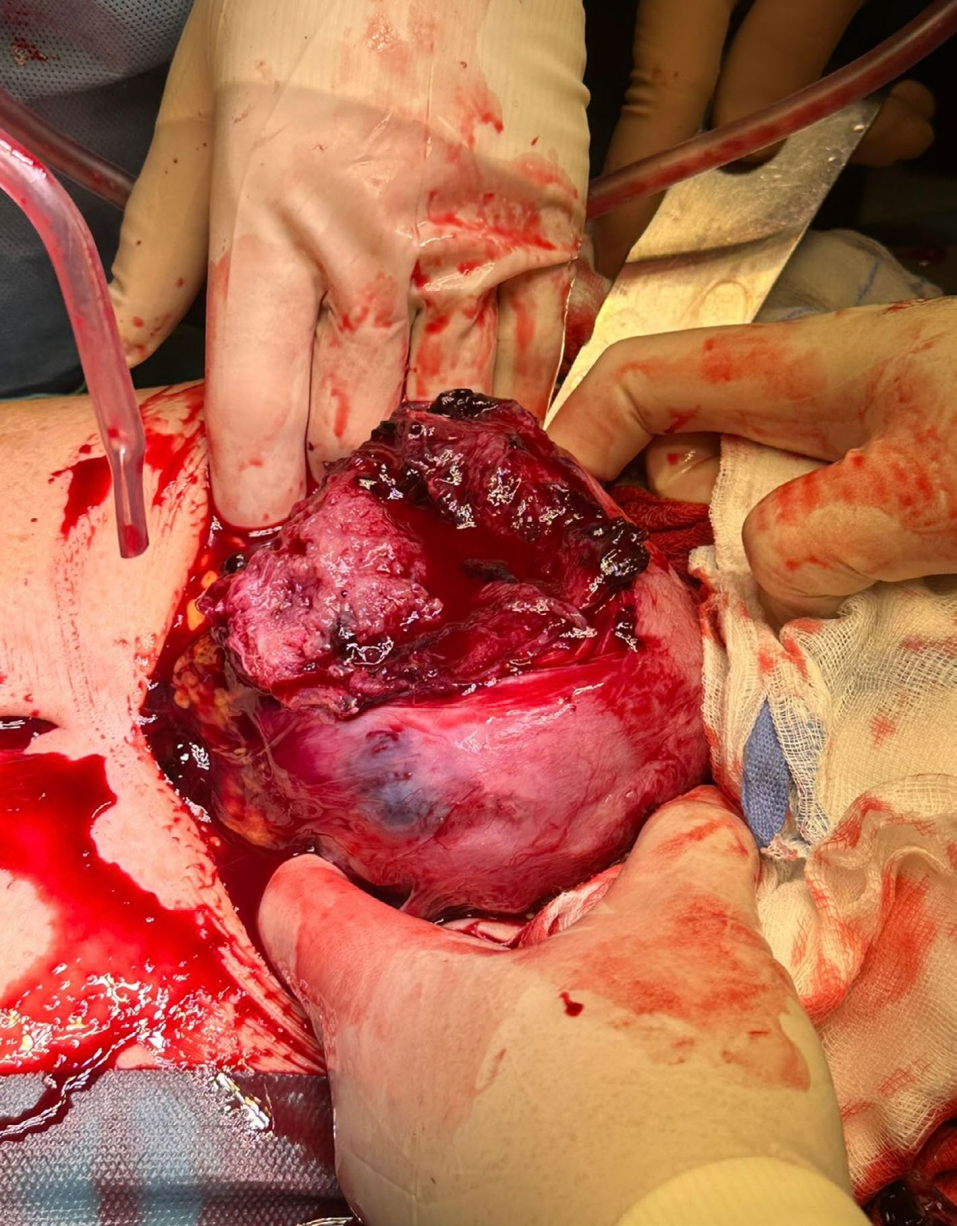
Uterine rupture 10 cm in size and located on the uterine fundus.

Transabdominal ultrasound scan at the end of the procedure confirmed the presence of a fetal heartbeat. One hour after hospitalization, the patient was admitted to the operating room; preoperative transabdominal ultrasound scan showed the absence of fetal heartbeat. Subsequently, exploratory laparotomy was performed, confirming a 3000 mL hemoperitoneum. A 10 cm fundal uterine rupture with protruding amniotic sac was present (Figures 2, 3, 4).

The surgeon hence proceeded to perform amniorrhexis, fetal extraction and manual removal of the placenta. Important adhesions between the posterior uterine wall and the intestine and between ovaries and uterus were described: adhesiolysis was performed. The uterus appeared fibromatous with areas of adenomyosis and a reduced consistency. The patient's uterus was finally surgically repaired with simple interrupted double layer suture. The total blood loss was 3200 mL.

The woman was then admitted to the intensive care unit (ICU) for 2 days due to the hemorrhagic shock and a total of 1250 mL of concentrated red blood cells and 750 mL of fresh frozen plasma were transfused. Further pharmacologic agents including antibiotics—Cefoxitin 3 × 1000 mg i.v.—as well as enoxaparin 4.000 units were administered. The patient recovered well and was discharged 7 days after surgery.

The second is a clinical case of a 32‐year‐old woman who had undergone laparoscopic myomectomy 23 months before conception. An intramural myoma 6 cm in diameter was located on the posterior wall of the uterus. The uterine cavity was not opened and the surgery was reported to be uncomplicated. The patient conceived spontaneously 23 months later. The course of the pregnancy was uncomplicated. The ultrasound scans of the fetus, placenta and uterus were normal. At 35 weeks of pregnancy, the fetal presentation was podalic, hence the patient received counseling about the mode of delivery: due to the previous myomectomy, the woman was not eligible for external cephalic version; thus, in case of spontaneous cephalic version, the patient could have been admitted to a trial of labor.

The woman presented at 36^+3^ weeks' gestation to the ED for irregular uterine contractions (1 uterine contraction every 10–15 min). On hospital presentation, vital signs were normal (arterial blood pressure 110/70 mmHg, heart rate 81 beats/min), transabdominal ultrasound evidenced a single fetus in breech presentation, with regular fetal heart rate. Nasopharyngeal swab for SARS‐CoV‐2 detection performed upon admission was negative and hemoglobin was 11.9 g/dL. The obstetric visit showed a closed, posterior cervix, with medium consistency and a 80% effacement. Cardiotocography (CTG) upon admission was reactive, with normal baseline variability and no decelerations, showing a fetal heart rate 140 beats/min.

Three hours after admission, transabdominal ultrasound confirmed a regular fetal heartbeat, the obstetric visit was unvaried.

On physical examination, the abdomen was soft, no rebound tenderness was identified. The uterus was palpable and mildly contracted. The uterus was palpable, but irregular uterine contractions were still present (1 uterine contraction every 10–15 min): the patient was thereby hospitalized. One hour after hospitalization, the patient reported a prolonged uterine contraction, and the transabdominal ultrasound check evidenced fetal bradycardia.

An immediate cesarean section was performed, showing a massive hemoperitoneum which was promptly drained. After fetal extraction and manual removal of the placenta, a close uterine inspection was performed, showing a 15 cm uterine rupture involving the posterior wall. The patient's uterus was finally surgically repaired with continuous triple layer suture. The total blood loss was 3000 mL. A girl was born alive, weighing 2960 g, APGAR score: 4, 8. Because of intraperitoneal bleeding the patient needed intraoperative transfusion of 4 × 250 mL of erythrocyte concentrate.

Further pharmacologic agents including antibiotics—Cefoxitin 3 × 1000 mg i.v.—as well as enoxaparin 4.000 units were administered. The patient was discharged in good condition on the sixth day after surgery.

The third is a clinical case of a 28‐year‐old woman who had undergone laparoscopic myomectomy 2 years before conception. Detailed documentation about the surgery was not available. The patient reported that the uterine cavity was opened to remove an intramural myoma of 6 cm in diameter located on the left anterolateral wall of the uterus. The surgery was reported to be uncomplicated by the patient. She conceived spontaneously 2 years later, and the course of the pregnancy was uncomplicated. The ultrasound scans of the fetus, placenta and uterus were normal.

The patient presented at 31^+0^ weeks' gestation to the ED for abdominal pain. On hospital presentation, vital signs were normal, no abnormal uterine bleeding was identified and fetal movements were well perceived.

On physical examination, the abdomen was soft but diffusely tender on deep palpation. The uterus was palpable and normotonic. No rebound tenderness was identified.

A transvaginal ultrasound scan showed a cervix >40 mm with free fluid in Douglas space, while transabdominal ultrasound scan evidenced a single fetus with normal heart rate and a growing blood clot at the uterine fundus. Nasopharyngeal swab for SARS‐CoV‐2 detection performed upon admission was negative.

**FIGURE 5:**
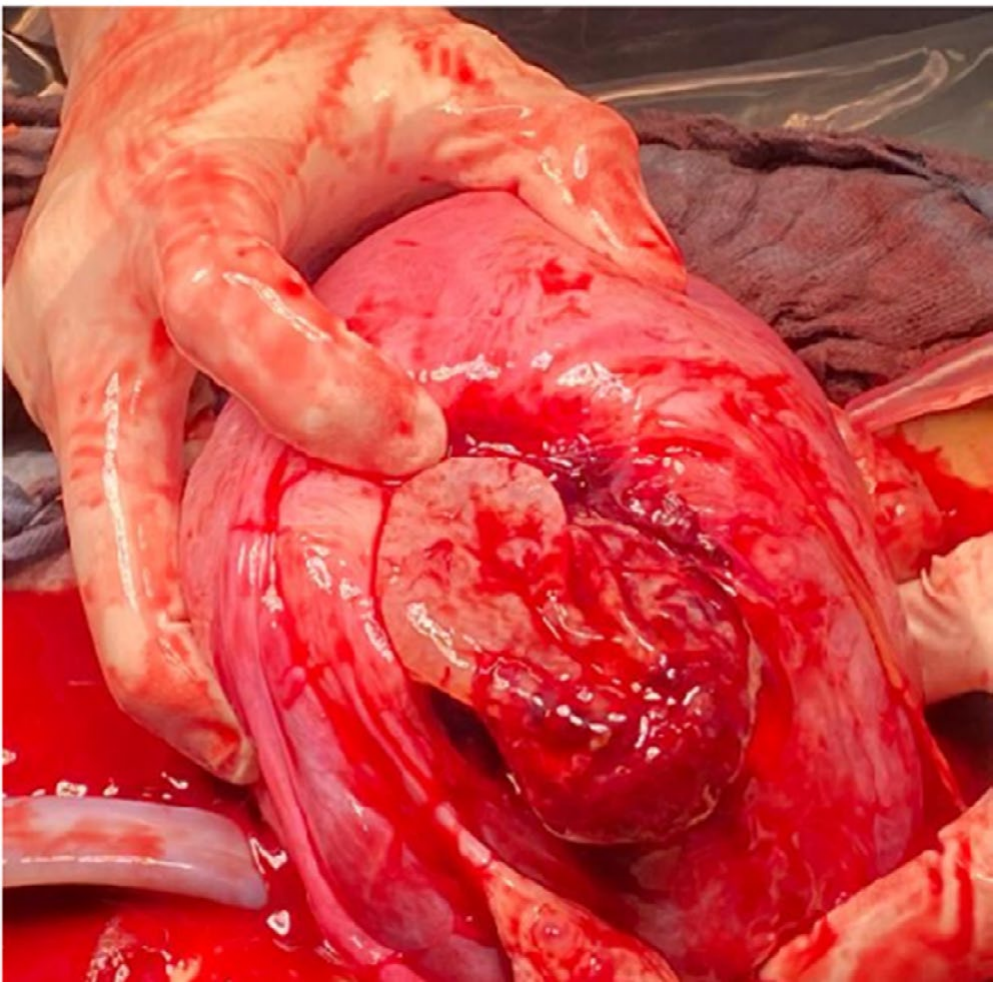
Uterine rupture identified during exploratory laparotomy.

**FIGURE 6:**
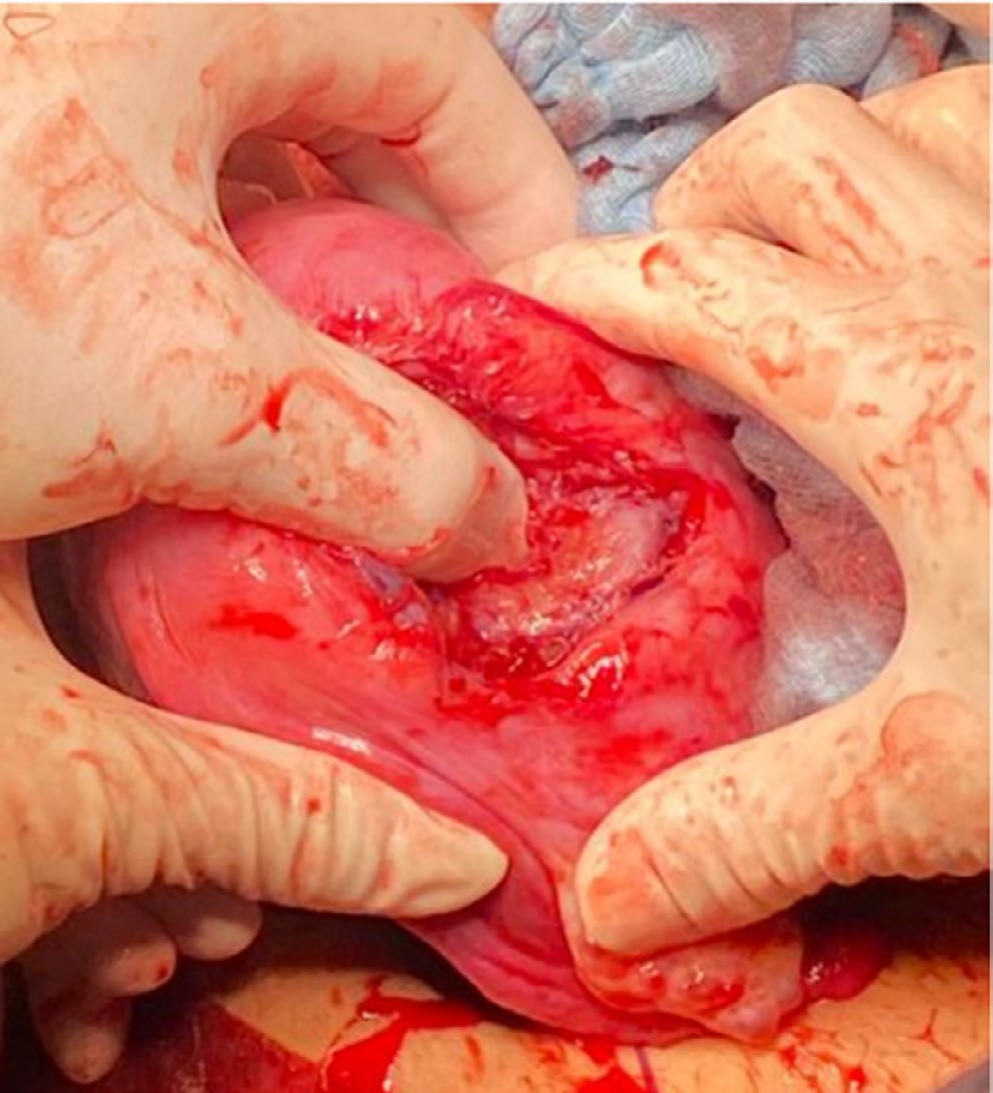
The myometrial defect reached the endometrial cavity. It was 7 cm in size and located on the left posterolateral uterine wall.

The patient was admitted to the operating room for exploratory laparotomy, confirming a massive hemoperitoneum. A 7 cm uterine rupture with protruding amniotic sac was present in the left posterolateral uterine wall (Figures 5 and 6). The surgeon hence proceeded to perform hysterotomy, amniorrhexis, fetal extraction of a fetus alive and vital, and manual removal of the placenta. The patient's uterus was surgically repaired with double layer suture.

The total blood loss was 1800 mL. A total of four units of packed red blood cells and two units of fresh frozen plasma were transfused. The patient recovered well and was discharged 7 days after surgery.

## 3 METHODS

### 3.1 Literature review

This study aimed to evaluate the incidence of spontaneous uterine rupture in pregnancy after myomectomy in women out of labor and to study characteristics of myomectomy thought to influence uterine rupture, such as the surgical technique (laparoscopy vs. laparotomy), the location of myoma and the use of diathermy cautery.

### 3.2 Information sources and search strategy

We obtained all articles related to our topic from the international electronic bibliographic databases PubMed/MEDLINE and Cochrane. The articles were identified using a combination of MeSH terms including the keywords “uterine rupture,” and “myomectomy.” The search was limited to studies reported in English and Italian. We did not employ temporal or publication status limits to restrict our search. Once an article was considered relevant, the full text was retrieved. The references included in the selected articles were also reviewed for related citations. Data collection and analysis were performed between December 2024 and January 2025.

### 3.3 Study selection and data retrieval

An independent researcher screened the titles and abstracts obtained to select the most relevant articles. The researcher selected the final studies to be included in the review after applying the eligibility criteria. Studies were considered eligible for review if they investigated clinical and surgical presentation in obstetric patients with a diagnosis of out of labor uterine rupture after myomectomy. Cases of uterine dehiscence were excluded. The following data were collected: authors, year of publication, maternal age at diagnosis, gestational age (GA) at diagnosis, obstetric history, and mode of delivery.

Statistical analysis was performed using the chi‐square test or the Fisher exact test. Confidence intervals were calculated using the Wilson intervals score. Statistical significance was set at *P* < 0.05.

## 4 RESULTS

A total of 28 studies reporting pregnancies after prior myomectomy, resulting in 3.502 viable (≥24 weeks) deliveries, were reviewed. Uterine rupture occurred in 21 cases, with an overall incidence of 0.6%. A total of 14 uterine rupture (0.4% of deliveries) occurred after laparoscopic procedures and seven (0.2% of deliveries) after open myomectomy.

Table 1 presents the 28 studies reviewed with information about number of pregnancies, deliveries, a trial of labor after myomectomy (TOLAM) and uterine rupture reported.

**TABLE 1:** Studies reporting pregnancies after myomectomy.

Author	Year	Country	LPS/LPT	Pregnancies	Deliveries	TOLAM	UR^a^	UR in labor
Obed & Omigbodun^5^	1996	Nigeria	NA	412	373	NA	1 (0.27%)	1
Darai et al.^6^	1997	France	LPS	25	12	NA	0	
Campo et al.^7^	1999	Italy	LPS	13	11	NA	0	
Nezhat et al.^8^	1999	USA	LPS	42	28	NA	0	
Ribeiro et al.^9^	1999	USA	LPS	18	14	NA	0	
Dubuisson et al.^10^, ^b^	2000	France	LPS	145	100	72	3 (3%)	0
Seinera et al.^11^	2000	Italy	LPS	65	56	18	0	
Seracchioli et al.^12^	2000	Italy	30 LPS 33 LPT	63	47 (27/47 LPT; 20/47 LPS)	22	0	
Dessolle et al.^13^	2001	France	LPS	44	34	30	0	
Rossetti et al.^14^	2001	Italy	LPS	21	15	NA	0	
Soriano et al.^15^	2003	France	88 LPS 18 LPT	54	38 (34/38 LPS; 2/48 LPT)	NA	0	
Landi et al.^16^	2003	Italy	LPS	76	57	31	0	
Campo et al.^17^	2003	Italy	LPS/LPT	29	25	NA	0	
Paul et al.^18^	2006	India	LPS	141	106		0	
Seracchioli et al.^19^	2006	Italy	LPS	158	106	44	0	
Sizzi et al.^20^	2007	Italy	LPS	386	309	NA	1 (0.32%)	0
Palomba et al.^21^	2007	Italy	36 LPS 26 mini‐LPT	62	54	35	0	
Makino et al.^22^	2008	Japan	LPS	109	109	72	0	
Kelly et al.^23^	2008	England	1 LPS 92 LPT	93	93 (92/93 LPT; 1/93 LPS)	72	1 (1.08%)	
Kumakiri et al.^24^	2008	Japan	LPS	221	111	74	0	
Lonnerfors et al.^25^	2011	Sweden	LPS	18	10	6	0	
Pitter et al.^26^	2013	USA	LPS	127	92	NA	1 (1.09%)	1
Kim et al.^27^	2013	Korea	340 LPS 75 LPT	66	53 (44/53 LPS) (9/53 LPT)	6	0	
Bernardi et al.^28^	2014	Germany	LPS	55	39	21	4 (10.25%)	2
Tian et al.^29^	2015	Cina	81 LPS 57 LPT	141	106 (61/106 LPS; 45/106 LPT)	NA	0	
Koo et al.^30^	2015	Korea	LPS	523	455	NA	3 (0.66%)	0
Ordàs et al.^31^	2022	Spain	36 LPS 23 LPT	59	52 (31/52 LPS; 21/52 LPT)	29	0	
Komatsu et al.^32^	2023	Japan	596 LPS 401 LPT	997	997 (596/997 LPS; 401/997 LPT)	290	7 (0.70%)	NA

Abbreviations: LPS, laparoscopy; LPT, laparotomy; NA, not available; TOLAM, trial of labor after myomectomy; UR, uterine rupture.

^a^ The percentage presented in the table represents the incidence of uterine ruptures as a proportion of the total number of deliveries.

^b^ One of the UR reported by Dubuisson was excluded because it was thought to be related to a subsequent surgical procedure other than myomectomy (a tubocornual anastomosis prior to pregnancy). Additionally, the UR in Obed & Omigbodun, Sizzi et al., and Pitter et al. were excluded from this analysis since there were no labor details available.

Among these studies, the ones that reported detailed data about a TOLAM and related pregnancy outcomes were further evaluated (Table 2).

**TABLE 2:** Rate of uterine rupture in 12 studies that reported detailed data about TOLAM.

Author	Year	Pregnancies	Pregnancy after myomectomy	Deliveries (≥24 weeks)	UR before labor	TOLAM	UR in TOLAM	Overall incidence of UR
Dubuisson et al.^10^	2000	145	All subsequent	100	2/100 (2%)	72/100	0/72	2/100
Seracchioli et al.^12^	2000	63	Not specified	47	0/47	22/47	0/22	0/47
Dessolle et al.^13^	2001	44	All subsequent	34	0/34	30/34	0/30	0/34
Serracchioli et al.^19^	2006	158	All subsequent	106	0/106	44/106	0/44	0/106
Palomba et al.^21^	2007	62	First pregnancy only	54	0/54	35/54	0/35	0/54
Makino et al.^22^	2008	109	Not specified	109	0/109	72/109	0/72	0/109
Kelly et al.^23^	2008	93	All subsequent	93	1/93 (1.1%)	72/93	0/72	1/93
Kumakiri et al.^24^	2008	221	First pregnancy only	111	0/111	74/111	0/74	0/111
Lonnerfors et al.^25^	2011	18	All subsequent	10	0/10	6/10	0/6	0/10
Kim et al.^27^	2013	66	Not specified	53	0/57	6/53	0/6	0/57
Bernardi et al.^28^	2014	55	All subsequent	39	2/39 (5.1%)	21/39	2/21	4/39
Ordàs et al.^31^	2022	59	All subsequent	52	0/52	29/52	0/29	0/52
Total		1.093		808	0.6% (5/808)	483/808	0.4% (2/483)	0.87% (7/808)

Abbreviations: TOLAM, trial of labor after myomectomy; UR, uterine rupture.

A total of 1.093 pregnancies were reported in these 12 studies, resulting in 808 ≥24‐week deliveries: 59.8% (*n* = 483/808) experienced a TOLAM, while 40.2% (*n* = 325/808) underwent cesarean delivery before labor (Table 2).

In the group of the 808 ≥24‐week deliveries, seven cases of uterine rupture were identified. The overall incidence of uterine rupture was 0.87%. The incidence of uterine rupture was 0.6% (*n* = 5/808) prior to the onset of labor, whereas it was 0.4% (*n* = 2/483) in the group who experienced a TOLAM; the uterine ruptures occurred at 37 and at 40 weeks. The difference of incidences of uterine rupture before or during labor was not statistically significant (*P* = 0.993). Cases of uterine rupture that occurred before labor did not differ from cases that occurred during labor regarding obstetrical and surgical available findings.

Many authors have discussed risk factors for uterine ruptures or dehiscence during pregnancies after myomectomy. The main risk factors are listed in Table 3. Among the selected studies, 10 reported adequate information regarding the surgical technique, hemostasis and the number of the layers of the performed sutures. Within the total cohort of 21 uterine rupture cases analyzed, 14 occurred in women who had prior laparoscopic myomectomy, and seven in patients who had a prior laparotomic myomectomy. The mean gestational age at uterine rupture is 31.66 gestational weeks. The incidence of uterine rupture was 0.2% after abdominal myomectomy and 0.4% after laparoscopic myomectomy. 21 uterine ruptures occurred at or before 36 weeks. Maternal and neonatal outcomes were reported to be favorable, except in one case which ended in twin fetal death. The presence of intramural myomas was more common in the uterine rupture group. Hemostasis was obtained using bipolar current and suture in eight (66.6%) cases, and monopolar current and suture was used in three (25%) cases. In one case this information was not available. The uterine wall was sutured with one layer in four women (33.3%) and with two layers in the other eight women (66.6%). When evaluated in detail, by specific type of hemostasis, either bipolar current, monopolar current or suture, the type of hemostasis was not a significant factor for uterine rupture.

**TABLE 3:** Characteristics of ≥24‐week women who experienced UR.

Author	LPS/LPT	Gestational age	Cavity entry	Suture layers	Hemostasis	Myomas (*n*)	Size (cm)	Type	UR in TOLAM
Dubuisson et al.^10^	LPS	32	No	1	BP	1	3	IM	No
Dubuisson et al.^10^	Mini‐LPT^a^	25	No	2	NA	1	8	IM	No
Sizzi et al.^20^	LPS	33	No	≥2	MP	10	10	NA	Yes
Kelly et al.^23^	LPS	36	No	1	MP	1	2.5	IM	No
Pitter et al.^26^	LPT	33	No	≥2	MP	10	10	NA	Yes
Bernardi et al.^28^, ^b^	LPS	24 (twin)	N/A^c^	2	BP	2	5	IM	No (vehicle accident)^d^
Bernardi et al.^28^	LPS	37	N/A^c^	2	BP	1	6	IM	No
Bernardi et al.^28^	LPS	40	N/A^c^	2	BP	3	5	IM	Yes
Bernardi et al.^28^	LPS	30	N/A^c^	2	BP	2	5	IM	Yes
Koo et al.^30^	LPS	37	No	2	BP	1	5	IM	No
	LPS	32	No	1	BP	1	5	SS	No
	LPS	21	No	1	BP	1	7	SS	No

Abbreviations: BP, bipolar current; IM, intramural; LPS, laparoscopy; LPT, laparotomy; MP, monopolar current; NA, not available; SS, subserosal; TOLAM, trial of labor after myomectomy; UR, uterine rupture.

^a^ Performed in another institution.

^b^ Two uterine cavities were entered, but it is not known from which of the four cases from this series.

^c^ No information available about cavity entry.

^d^ Patient went into preterm labor after trauma.

## 5 DISCUSSION

Our review of the literature, including 28 studies with 3.502 pregnancies after prior myomectomy, shows an overall incidence of uterine rupture after myomectomy of 0.6%, comparable with those reported in other reviews. Gambacorti‐Passerini et al. reported an incidence of 0.93%.^33^

Our review confirmed that the incidence of uterine rupture is very low, 0.4%, in the group of women who experienced a TOLAM.

In particular, the difference of incidences of uterine rupture before or during labor was not statistically significant. Therefore, uterine rupture may not be significantly influenced by a TOLAM and so this option could be considered in pregnant women as feasible and relatively safe.

Some authors^33^ raised the doubt that this result was influenced by the fact that some women underwent a planned cesarean delivery before 39 weeks, thus preventing some uterine ruptures. However, the incidence of uterine rupture was higher in women who experienced a uterine rupture without labor than during a TOLAM.

## 6 CONCLUSION

The first crucial point is that the available data in the literature is limited.

Moreover, the studies analyzed in this review lack critical information, such as the age of the women and whether they were affected by adenomyosis, which complicates data comparison.

Finally, the most remarkable finding is that uterine rupture in patients with a history of previous myomectomy does not occur more frequently during labor.

A randomized study of a TOLAM versus planned cesarean at term would be useful to confirm our findings by providing more evidence.

## AUTHOR CONTRIBUTIONS

V.S. had the original idea and designed the present article. A.B. collected, prepared, analyzed, and interpreted the data for the present study with supervision from V.S., E.C. and S.B.

A.B. wrote the manuscript. All authors were involved in editing the document and approved the final version before publication.

## CONFLICT OF INTEREST STATEMENT

The authors have no conflicts of interest to declare.

## Data Availability

Data sharing is not applicable to this article as no new data were created or analyzed in this study.
